# An improved genetic system for detection and analysis of protein nuclear import signals

**DOI:** 10.1186/1471-2199-8-6

**Published:** 2007-01-25

**Authors:** Kris S Marshall, Zhiying Zhang, Jennifer Curran, Stephanie Derbyshire, Joe S Mymryk

**Affiliations:** 1 Departments of Microbiology & Immunology and Oncology, The University of Western Ontario, London Regional Cancer Program, London, Ontario, N6A 4L6, Canada; 2 Gemin X Biotechnologies Inc. 3576 Avenue du Parc, suite 4310, Montreal, Quebec, H2X 2H7, Canada

## Abstract

**Background:**

Nuclear import of proteins is typically mediated by their physical interaction with soluble cytosolic receptor proteins via a nuclear localization signal (NLS). A simple genetic assay to detect active NLSs based on their function in the yeast *Saccharomyces cerevisiae *has been previously described. In that system, a chimera consisting of a modified bacterial LexA DNA binding domain and the transcriptional activation domain of the yeast Gal4 protein is fused to a candidate NLS. A functional NLS will redirect the chimeric fusion to the yeast cell nucleus and activate transcription of a reporter gene.

**Results:**

We have reengineered this nuclear import system to expand its utility and tested it using known NLS sequences from adenovirus E1A. Firstly, the vector has been reconstructed to reduce the level of chimera expression. Secondly, an irrelevant "stuffer" sequence from the *E. coli *maltose binding protein was used to increase the size of the chimera above the passive diffusion limit of the nuclear pore complex. The improved vector also contains an expanded multiple cloning site and a hemagglutinin epitope tag to allow confirmation of expression.

**Conclusion:**

The alterations in expression level and composition of the fusions used in this nuclear import system greatly reduce background activity in β-galactosidase assays, improving sensitivity and allowing more quantitative analysis of NLS bearing sequences.

## Background

The proper function of many proteins requires that they are targeted to their correct sub-cellular compartments via specific localization signals. Nuclear import of many cellular and viral proteins is typically mediated by their physical interaction with soluble cytosolic receptor proteins via nuclear localization signals (NLS) [1]. A canonical NLS is characterized by a short single stretch of basic amino acids, as exemplified by the NLS sequence of the large T antigen of the simian virus 40 (PKKKRKV) [2]. These monopartite signals generally contain at least three basic amino acids (B) with a consensus sequence fitting B_4_, P(B_3_X), PXX(B_3_X), B_3_(H/P), where P is proline, H is histidine, X is any amino acid and letters in parentheses can be in any order [1]. Alternatively, bipartite NLSs contain two short stretches of basic amino acids separated by a non-conserved sequence, as found in the cellular nucleoplasmin protein (KRPAATKKAGQAKKKK) [3].

Proteins containing NLSs are imported into the nucleus by interacting in the cytosol with members of the importin α family of NLS receptors (also known as karyopherin α). Subsequent heterodimerization of importin α with importin β (also known as karyopherin β) and interaction with components of the nuclear pore complex (NPC) leads to translocation through the nuclear envelope in a GTP dependent fashion. Inside the nucleus, the importin α-substrate complex dissociates from importin β and the substrate subsequently dissociates from importin α [4]. The yeast *Saccharomyces cerevisiae *express a single importin α, Srp1 [5], whereas multiple isoforms have been isolated in higher eukaryotes. Each isoform of importin α has distinct substrate specificities, suggesting that this may contribute to the regulation of nuclear import [1]. Despite the fact that many thousands of cellular proteins are transported into the nucleus, very few NLSs have been characterized in detail, and existing work has been biased towards classical monopartite or bipartite signals. As nuclear transport regulates, at least in part, an extraordinarily diverse range of cellular processes including cell cycle, signal transduction, apoptosis and circadian rhythm, the identification of mechanisms regulating nuclear import is a valuable area of investigation.

The largest protein encoded by the human adenovirus type 5 (Ad5) early region 1A (*E1A*) gene contains 289 amino acid residues (289R). A highly related 243 amino acid (243R) protein is produced by alternative splicing [6]. E1A is present in roughly equal amounts in the nucleus and cytoplasm and both the 289R and 243R proteins contain a well characterized monopartite NLS (KRPRP) located at their C-terminus [7]. This NLS mediates nuclear import *in vitro *and *in vivo *and shows a distinct preference for human importin α3 *in vitro *[8]. A second non-conventional NLS with the consensus sequence FV(X)_7–20_MXSLXYM(X)_4_MF, spans residues 142–182 and is unique to the larger 289 residue E1A protein [9,10]. This sequence does not resemble other known NLSs and appears to be regulated developmentally [11]. At least one other non-canonical NLS is present elsewhere within the E1A protein, as residues 23–120 of E1A have been reported to be sufficient to mediate nuclear accumulation via an unknown pathway in microinjected *Xenopus laevis *oocytes [12]. Another study, which used indirect immunofluorescence to examine the subcellular localization of 243R E1A in virally infected baby rat kidney cells also demonstrated that the N-terminal region spanning residues 30–85 were involved in nuclear localization. In addition, this latter study confirmed that the well characterized C-terminal NLS was necessary for efficient nuclear localization and further suggested a role for a region encompassing residues 186–220 in infected cells [13]. It is not currently known why E1A contains so many different NLSs. However, it is likely that the actions of E1A, like those of some cellular proteins [14], are closely regulated through modulation of the level of nuclear import. As the E1A proteins possess such an array of non-conventional nuclear import functions, they provide an excellent model system to identify and characterize novel mechanisms that contribute to nuclear localization.

The lack of clearly defined and consistent NLS consensus motifs makes it difficult to predict their presence in a protein of interest. Until recently, the only practical way to identify a functional NLS was by microinjecting or otherwise expressing the test protein in eukaryotic cells, forming heterokaryons or using *in vitro *transport systems. As an alternative, an easier and more sensitive method that exploited yeast genetics was devised to detect an active NLS, which is based on the expression of the test protein fused to a modified LexA DNA binding domain (DBD) and the Gal4 transcriptional activation domain (AD) in the yeast *S. cerevisiae *[15]. This transcription based assay relies on the ability of a functional NLS to allow the chimera to enter the yeast nucleus and activate transcription of a LexA responsive β-galactosidase or *HIS3 *reporter gene. In the absence of a functional NLS, the fusion protein is not efficiently imported into the nucleus, and is unable to activate transcription. As a result, this assay provides a simple qualitative measure of NLS function based on β-galactosidase activity assays or by monitoring yeast growth on medium lacking histidine respectively. This system is not limited to the identification of yeast NLSs, as the nuclear import apparatus is highly conserved between yeast and higher eukaryotic cells [1,15].

We have obtained this system and verified that Ad5 E1A actively functions to promote nuclear import under these conditions. However, the original system had two major problems that have now been corrected. A high level of background activity was initially observed, which was in part related to the high levels of test protein expression from the original multicopy pNIA vector. To solve this issue, we reconstructed the expression vector as a single copy yeast plasmid. The small size of the initial pNIA LexA fusion allows the chimera to passively diffuse into the nucleus, increasing the background activity. We have re-engineered the vector with an additional "stuffer" sequence that encodes a portion of the *E. coli *maltose binding protein that contains no NLS activity. This increases the size of the chimera well above the commonly accepted 50 kDa passive diffusion limit of the nuclear pore complex [1] and greatly reduces background activity in the β-galactosidase assays. The improved vector also contains an expanded multiple cloning site that simplifies cloning of target sequences and a hemagglutinin (HA) epitope tag to allow confirmation of expression.

Using this improved system, we demonstrate that it faithfully detects all the known E1A NLS functions in a quantitative fashion. Thus, this improved system may aid in the identification and characterization of novel NLS activities.

## Results and discussion

### Construction and evaluation of an improved vector for detecting nuclear import

A simple transcription based assay in yeast for detecting nuclear import has been described [15]. Using this system, we evaluated nuclear import conferred by fusing the full length Ad5 289R E1A protein or the N-terminal 82 amino acids (1–82) in frame to the modified LexA DBD-Gal4 AD chimera (Fig. 2). Although a substantial increase in β-galactosidase activity was detected with E1A 1–82 as compared to the empty pNIA vector, surprisingly little change was observed with the full length 289R E1A protein or the well studied SV40 large T antigen NLS. Based on these results, we pursued a series of modifications to improve the quantitative functionality of this system and its general utility.

**Figure 1:**
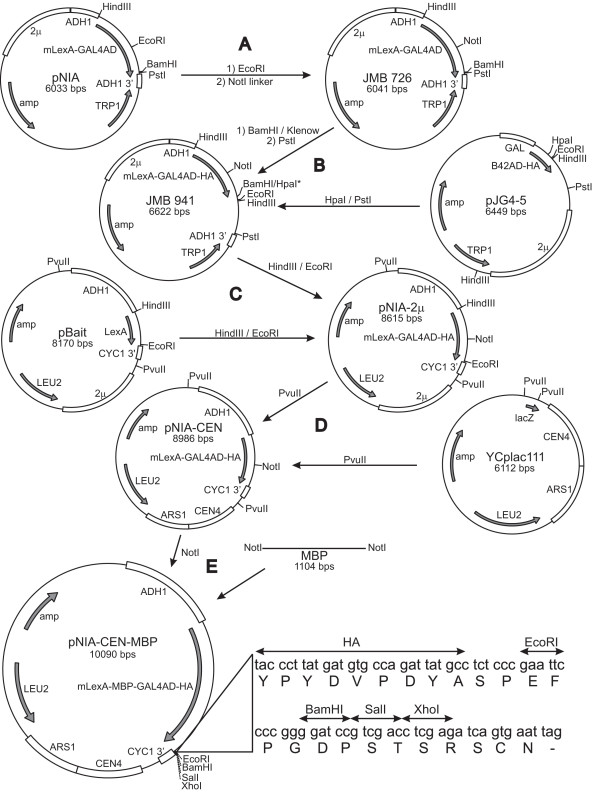
**Schematic of the construction of pNIA derivatives used in this study**. Restriction enzyme sites used for each cloning step, relevant markers and features of each plasmid are indicated. The construction scheme is detailed in the Materials and Methods section. The sequence of the HA tag and the unique restriction enzyme sites in the polylinker of pNIA-CEN-MBP plasmid is shown at the bottom right. * indicates site that is destroyed

**Figure 2:**
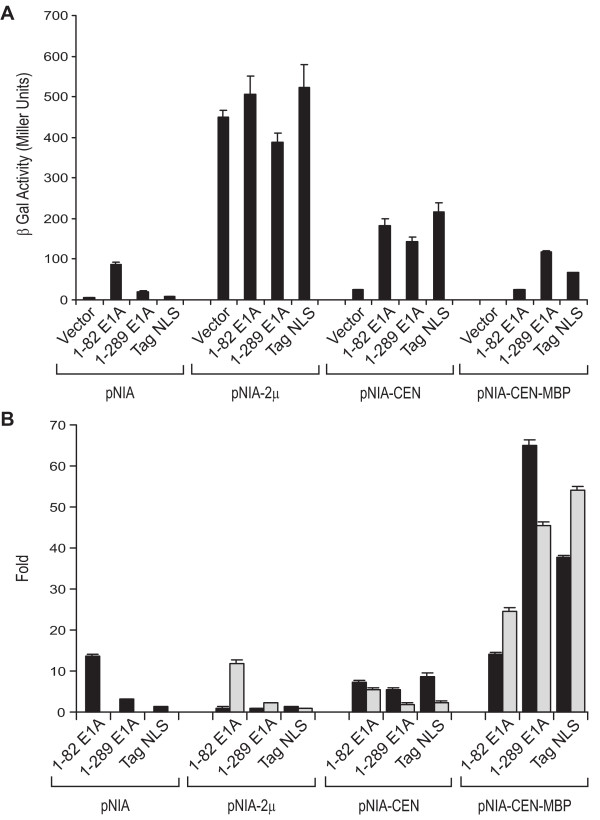
**Analysis of nuclear import in yeast as measured by each subsequent generation of vector**. A) Absolute values of β-galactosidase reporter activity with the indicated constructs (means ± SD, n = 3). B) Relative values of β-galactosidase reporter activity. In each case, the corresponding empty control vector was arbitrarily set at a value of 1. Lightly shaded bars indicate values normalized to protein expression, whereas bars shaded in black represent the original ratios (means ± SD, n = 3). Tag NLS – SV40 large T antigen NLS

**Figure 3:**
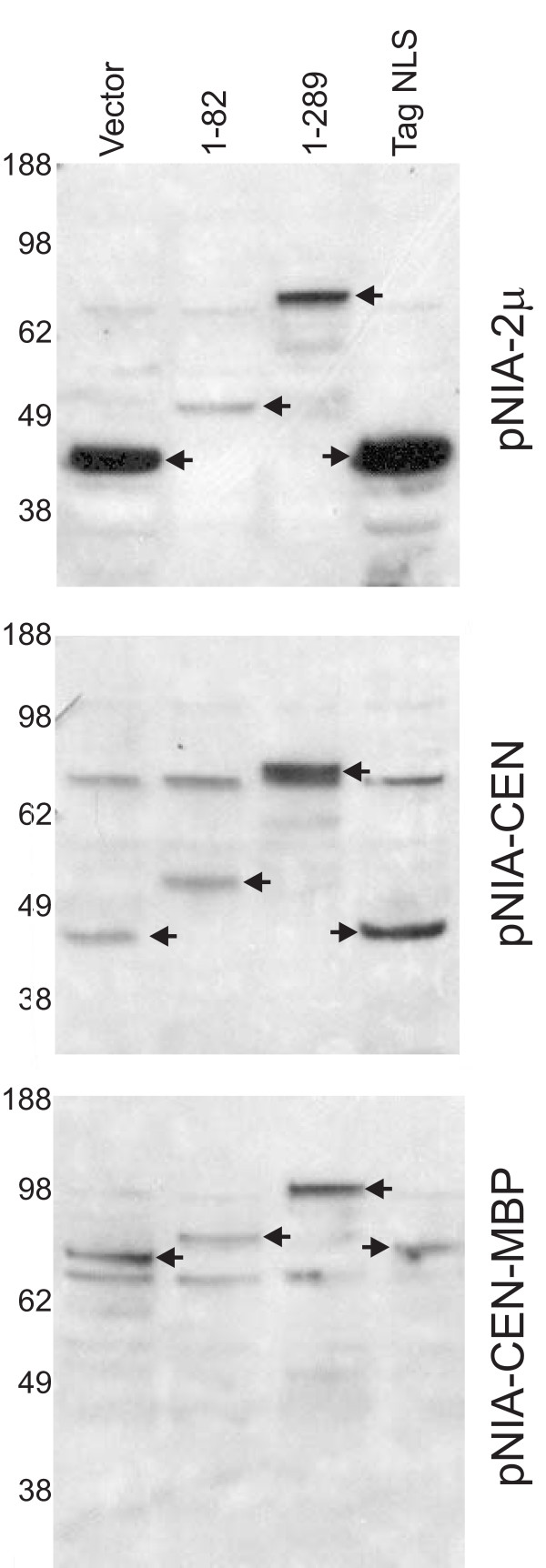
**Western blot analysis of the expression of the various chimeras**. Extracts from L40 strain yeast transformed with the indicated expression constructs were analyzed by Western blot to ensure expression of the HA tagged fusions. All samples were prepared and analyzed concurrently. 30 μg of extract was loaded per lane. Molecular weight markers are indicated on the left. Bands of interest are indicated with arrows. Top – pNIA-2μ, Middle – pNIA-CEN, Bottom – pNIA-CEN-MBP. Tag NLS – SV40 large T antigen NLS

As described in the Materials and Methods and illustrated in Fig. 1, we first reconstructed the pNIA LexA DBD-Gal4 AD into another multicopy plasmid backbone originally derived from YEplac181 [16]. This yielded pNIA-2μ, which has a known nucleotide sequence, contains an improved polylinker and has an HA epitope tag to allow convenient detection of expression of the LexA fusion. However, this vector contains the entire *ADH1 *promoter, which is substantially more active than the truncated *ADH1 *promoter in the original pNIA. Tests with the same candidate NLSs in pNIA-2μ showed a uniformly high level of activity for all constructs, including the empty vector (Fig. 2). This was likely caused by the very high level of expression of the LexA DBD fusions from the strong promoter in a multicopy plasmid. Indeed, Western blot analysis of the expression levels of the various fusions using an anti-HA antibody confirmed abundant expression, with the exception of E1A 1–82 (Fig. 3). Recalculation of the nuclear import based on relative levels of protein expression suggested that E1A 1–82 continues to display import activity above that of the corresponding empty vector (Fig. 2B).

To reduce the high level of protein expression from pNIA-2μ, we shifted the entire expression cassette into the single copy YCplac111 vector [16] and tested the same candidate NLSs in pNIA-CEN. Western blot analysis with the HA tag confirmed that expression levels of the fusions were lower in yeast transformed with the single copy plasmid than the multicopy plasmid (Fig. 3). Again, E1A 1–82 was consistently expressed at a lower level than the other constructs. The background activity of the empty pNIA-CEN vector was dramatically lower than that of pNIA-2μ (Fig. 2A). Importantly, all candidate NLSs conferred a strong increase in β-galactosidase activity in pNIA-CEN, regardless of whether they are normalized to protein expression (Fig. 2B). These results clearly show that overexpression of the LexA DBD fusions by the multicopy plasmid obscures nuclear import.

It is commonly accepted that the nuclear pore complex has a passive diffusion limit of approximately 50 kDa [1]. The LexA DBD-Gal4 AD-HA fusion produced by the empty pNIA-2μ and pNIA-CEN vectors is 363 amino acids in size with a predicted molecular weight of about 40 kDa (Fig. 3). Thus, passive diffusion of the test fusion into the nucleus likely occurs and this could contribute to the relatively high background activity in the β-galactosidase assays. To overcome this issue, we inserted a "stuffer" fragment corresponding to a portion of the *E. coli *maltose binding protein (MBP) in frame between the LexA DBD and Gal4 AD to create pNIA-CEN-MBP. Analysis of this fragment of MBP with PSORT did not detect any sequences predicted to function as an NLS [4], and this was confirmed experimentally (Fig. 2). Insertion of this fragment increases the molecular weight of the test fusion to about 80 kDa (Fig. 3), well above the passive diffusion limit. All candidate NLSs confer a strong increase in β-galactosidase activity in pNIA-CEN-MBP. Importantly, the background activity of the empty vector decreased to nearly undetectable levels, yielding very large fold differences between the empty vector and vectors containing control NLSs (Fig. 2B). When compared with pNIA-CEN, these results suggest that passive diffusion of the smaller LexA DBD fusions obscures active nuclear import conferred by the candidate NLSs. Thus, the pNIA-CEN-MBP vector appears ideally suited for the analysis of short sequences that may function as NLSs. This is exemplified by the short SV40 large T antigen NLS, which showed the most dramatic increase in import activity between pNIA-CEN and pNIA-CEN-MBP (Fig. 2).

### Analysis of nuclear import conferred by various portions of E1A

**Figure 4:**
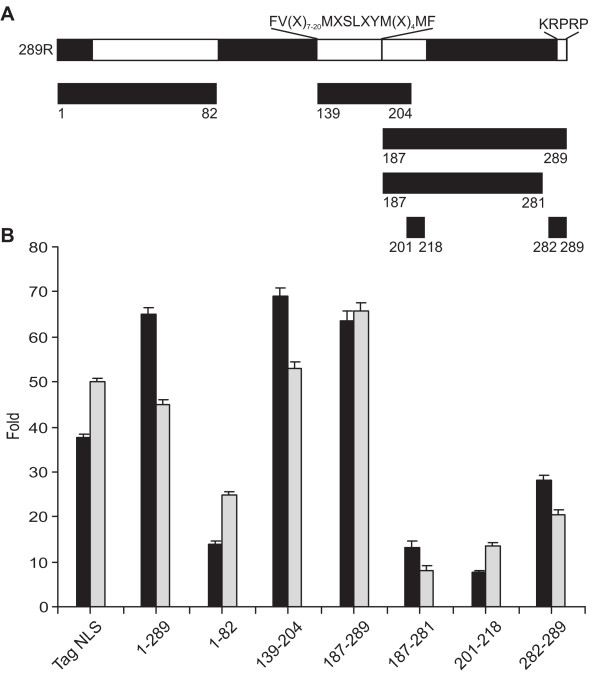
**Analysis of nuclear import mediated by various regions of E1A**. A) Map of the 289 residue human adenovirus type 5 E1A protein, previously identified regions known to confer nuclear import and constructs used in this study. The indicated amino acid numbers are inclusive. B) Effects of the various indicated E1A regions on nuclear import in yeast as measured with the β-galactosidase reporter. In each case, the corresponding empty control vector was arbitrarily set at a value of 1, whereas bars shaded in black represent the original ratios (means ± SD, n = 3). Lightly shaded bars indicate values normalized to protein expression.

As described in the introduction, E1A contains a canonical NLS with the sequence KRPRP spanning residues 285–289. In addition, a second non-conventional NLS with the consensus sequence FV(X)_7–20_MXSLXYM(X)_4_MF, spanning residues 142–182 has been described and a third activity is localized within residues 30–85. PSORT analysis [4] of E1A also predicts the presence of a second canonical NLS with the sequence RRPK spanning residues 205–208, which is located within a region previously identified as influencing nuclear localization of E1A in infected baby rat kidney cells [13]. Although the modified pNIA can detect the SV40 large T antigen NLS, and at least the N-terminal NLS in E1A (Fig. 2), it was unclear whether this yeast system can faithfully detect other NLSs that function in mammalian cells. To address this issue, we tested a number of fragments of E1A in pNIA-CEN-MBP to see if the various regions previously shown to confer nuclear import in a variety of other test systems were detected using this yeast system (Fig. 4). As previously shown in Fig. 2, full length 289R E1A or E1A 1–82 function to direct nuclear localization of the LexA chimera. In addition, E1A 139–204, which contains a developmentally regulated non-canonical NLS, also functions in the yeast system. Similarly, E1A 187–289, which encompasses the well characterized canonical NLS, as well as the PSORT predicted NLS, confers nuclear import. Deletion of this well characterized NLS, to generate E1A 187–281, greatly reduces import, suggesting that the major activity resides in the well characterized canonical NLS. In agreement, fusion of just this canonical NLS, contained within E1A 282–289, is sufficient to induce strong import of the chimera. Fusion of E1A 201–218, which contains a canonical NLS predicted by PSORT, exhibited the lowest level of import activity. As such, this signal may not function efficiently, although it is located within a region of E1A known to influence nuclear localization in infected cells [13]. Thus, the yeast nuclear import system appears quite versatile and is capable of recognizing all the known functional NLSs within E1A, whether they represent canonical or non-canonical sequences. Furthermore, this system may be sensitive enough to uncover weak import sequences. However, one obvious caveat of this technology, or any other approach that uses small fragments of proteins for identifying sequences directing nuclear import, is that these activities may not function in the context of the full length folded protein. In many cases, the effect of mutating the putative NLS on nuclear localization of the full length protein could be tested, although this is not feasible in proteins such as E1A that contain multiple nuclear localization signals.

## Conclusion

The availability of a simple and quantitative transcription based method for the identification and functional analysis of nuclear import in yeast provides a powerful alternative method for studying the complexities of nucleocytoplasmic transport. Although useful for analysis of individual proteins, the availability of this technology may allow high throughput analysis of nuclear import potential and could be readily applied to large scale screens of short random sequence to identify novel potential NLSs.

## Methods

### Plasmid construction

The construction of pNIA-2μ, pNIA-CEN and pNIA-CEN-MBP is illustrated in Fig. 1. Briefly, pNIA [15] was digested with EcoRI and ligated with a NotI linker to create JMB726 (A). JMB726 was digested with BamHI, blunt ended by fill in with the Klenow fragment of *E. coli *DNA polymerase I, and further digested with PstI. This fragment was ligated into a compatible HpaI-PstI digested pJG4-5 (Clontech, Mountain View, CA) containing the hemagglutinin (HA) epitope tag and polylinker to form JMB941 (B). A HindIII-EcoRI fragment from JMB941, encoding the mutant LexA-Gal4 activation domain-HA tag, was ligated into similarly digested pBAIT [17] to construct pNIA-2μ, which contains an expanded polylinker (C). pBAIT is a derivative of YEplac181 [16], which contains the *ADH1 *promoter and *CYC1 *terminator from pRS423ADH [18]. A PvuII fragment encoding the entire nuclear import expression cassette, including transcriptional promoter and terminator was transferred from pNIA-2μ into YCplac111 [16] to generate the single copy pNIA-CEN vector (D). Finally, an 1104 base pair stuffer fragment encoding a portion of the *E. coli *maltose binding protein was amplified by PCR and inserted in frame into the unique NotI site of pNIA-CEN to create pNIA-CEN-MBP, which expresses a 731 amino acid fusion with a molecular weight of 80.3 kDa (E). BamHI-PstI fragments corresponding to the full length 289R E1A protein or residues 1–82 were inserted in frame into pNIA. pNEA contains the SV40 large T antigen NLS inserted into the EcoRI site of pNIA [15]. EcoRI-SalI or EcoRI-XhoI fragments corresponding to the full length 289R E1A protein, residues 1–82, 139–204, 187–289, 187–282, 201–218, 282–289 or the SV40 large T antigen NLS were inserted into the corresponding sites of pNIA-2μ, pNIA-CEN or pNIA-CEN-MBP as indicated in the text.

### β-galactosidase assays

L40 strain yeast (Invitrogen, Carlsbad, CA) were transformed with pNIA or the derived plasmids by the lithium acetate procedure as described [19]. Cells were plated on glucose supplemented synthetic complete medium lacking either tryptophan or leucine and grown at 30°C. Transformed yeast colonies were used to inoculate 5 ml of glucose supplemented synthetic complete medium lacking either tryptophan or leucine and grown overnight at 30°C with agitation. Cells were collected by centrifugation at 4000 × g for 30 seconds. β-galactosidase assays were performed in triplicate as described previously [20].

### Western blot analysis of protein expression

Expression levels in yeast were determined by Western blot analysis. L40 strain yeast were transformed with pNIA or derivatives and plated on SC selective medium containing glucose. Colonies of transformed yeast were picked from each plate and used to inoculate 5 ml of liquid SC selective medium containing glucose, which were then grown in a cell culture rotator at 30°C overnight. Cells were harvested by centrifugation, washed with sterile distilled H_2_O, washed twice in 1 ml of buffer (20 mM HEPES pH 7.5), resuspended in 200 μl of buffer containing Complete Protease Inhibitor Cocktail (Roche Diagnostics, Laval, PQ) and transferred into fresh tubes containing 0.3 g of 425–600 micron acid-washed glass beads (Sigma-Aldrich, Oakville, ON). Cells were lysed by 3 cycles of vortexing for 3 minutes followed by 3 minutes of incubation on ice. After lysis, samples were centrifuged at 14,000 × g for 30 min. 200 μl of each supernatant were transferred to fresh microcentrifuge tubes, recentrifuged as above and the protein concentration of the supernatants was measured using Bio-Rad DC Protein Assay (Bio-Rad Laboratories, Mississauga, ON). 30 μg of protein from each extract were separated on NuPAGE 4–12% gradient SDS-polyacrylamide gels (Invitrogen, Carlsbad, CA) and transferred to a PVDF membrane (GE Healthcare Life Sciences, Piscataway, NJ). Western blot analyses were performed with the ECL Plus system (Amersham Pharmacia Biotech, Piscataway, NJ), using the 3F10 anti-HA rat monoclonal antibody (Roche Diagnostics, Laval, PQ) and goat anti-rat IgG horseradish peroxidase conjugated antibody (Pierce, Rockford, IL). Blots were processed concurrently and exposed to film for the same amount of time. Quantitation of protein expression was performed using ImageQuant (GE Healthcare Life Sciences, Piscataway, NJ).

## Authors' contributions

All experimental procedures were carried out by KM, ZZ, JC and SD. JM contributed to the design of the study. All authors read and approved the final manuscript.
